# Identification of Gene Signatures and Expression Patterns During Epithelial-to-Mesenchymal Transition From Single-Cell Expression Atlas

**DOI:** 10.3389/fgene.2020.605012

**Published:** 2021-01-28

**Authors:** Xiangtian Yu, XiaoYong Pan, ShiQi Zhang, Yu-Hang Zhang, Lei Chen, Sibao Wan, Tao Huang, Yu-Dong Cai

**Affiliations:** ^1^Clinical Research Center, Shanghai Jiao Tong University Affiliated Sixth People’s Hospital, Shanghai, China; ^2^Key Laboratory of System Control and Information Processing, Ministry of Education of China, Institute of Image Processing and Pattern Recognition, Shanghai Jiao Tong University, Shanghai, China; ^3^Department of Biostatistics, University of Copenhagen, Copenhagen, Denmark; ^4^CAS Key Laboratory of Computational Biology, Bio-Med Big Data Center, CAS-MPG Partner Institute for Computational Biology, Shanghai Institute of Nutrition and Health, Chinese Academy of Sciences, Shanghai, China; ^5^College of Information Engineering, Shanghai Maritime University, Shanghai, China; ^6^Shanghai Key Laboratory of PMMP, East China Normal University, Shanghai, China; ^7^School of Life Sciences, Shanghai University, Shanghai, China

**Keywords:** gene signature, expression pattern, epithelial-to-mesenchymal transition, single cell, classification

## Abstract

Cancer, which refers to abnormal cell proliferative diseases with systematic pathogenic potential, is one of the leading threats to human health. The final causes for patients’ deaths are usually cancer recurrence, metastasis, and drug resistance against continuing therapy. Epithelial-to-mesenchymal transition (EMT), which is the transformation of tumor cells (TCs), is a prerequisite for pathogenic cancer recurrence, metastasis, and drug resistance. Conventional biomarkers can only define and recognize large tissues with obvious EMT markers but cannot accurately monitor detailed EMT processes. In this study, a systematic workflow was established integrating effective feature selection, multiple machine learning models [Random forest (RF), Support vector machine (SVM)], rule learning, and functional enrichment analyses to find new biomarkers and their functional implications for distinguishing single-cell isolated TCs with unique epithelial or mesenchymal markers using public single-cell expression profiling. Our discovered signatures may provide an effective and precise transcriptomic reference to monitor EMT progression at the single-cell level and contribute to the exploration of detailed tumorigenesis mechanisms during EMT.

## Introduction

Cancer, which refers to abnormal cell proliferative diseases with systematic pathogenic potentials, is one of the leading threats to human health in the 21st century (McGuire, 2016). According to the statistics provided by the World Health Organization (WHO) and the Cancer Research United Kingdom organization, approximately 10 million people around the world died due to cancer in 2018, and 17 million people were diagnosed with cancer (McGuire, 2016). More than 20% (approximately four million) of these patients are diagnosed in China (McGuire, 2016; Yao et al., 2017). More than 10,000 people are diagnosed with cancer every day, implying the serious harm of cancer worldwide, especially in China.

Although cancer has been widely regarded as a deadly disease, patients with only primary sites can survive for a long time under tumor-burdening conditions (Barbaric et al., 2010; Huang et al., 2017; DeTroye et al., 2018). The final causes of patient death are usually cancer recurrence, metastasis, and drug resistance against continuing therapy (Fidler, 2003). Under these circumstances, the rate of tumor progression could be accelerated dramatically due to systematic pathogenic influences; no drugs can be used to control such malignant proliferative disease, which may lead to death. Epithelial-to-mesenchymal transition (EMT), which is the major transformation of tumor cells (TCs), is the prerequisite for pathogenic cancer recurrence, metastasis, and drug resistance; and EMT is one of the most significant cancer behaviors during pathogenesis (Rokavec et al., 2014; Chaffer et al., 2016; Shibue and Weinberg, 2017).

Early in the 1980s, EMT was recognized and confirmed to be a typical biological cellular transformation in embryogenesis but not in tumorigenesis (Kong et al., 2011; Das et al., 2019). EMT has been regarded as a specific biological progression for differentiation of multiple tissue subtypes, whose reverse progression is also known as mesenchymal-to-epithelial transformation (MET) (Das et al., 2019). In 2000, EMT was first confirmed to participate in cancer invasion and metastasis progression (Hanahan and Weinberg, 2000). EMT is one of the most substantially prerequisite for the formation of circulating TCs in the bloodstream, revealing the specific role of EMT during metastasis (Chaffer and Weinberg, 2011). In 2007, another independent study confirmed that EMT is involved in drug resistance against paclitaxel in ovarian carcinoma epithelial cell lines (Kajiyama et al., 2007), thereby validating the specific role of EMT during different stages of tumorigenesis. At present, EMT has been gradually confirmed to be a unique biological process that plays different functional roles during different tumorigeneses.

Epithelial-to-mesenchymal transition progression has been precisely regulated by various genes on different levels; scholars have attempted to identify the biomarkers of epithelial and mesenchymal TCs for a long time. According to recent publications, various biomarkers for monitoring EMT has already been identified including: cell-surface proteins: N-cadherin, cytoskeletal markers: FSP1 and α-SMA, extracellular matrix proteins: α1(I,III) collagens, transcription factors: Snail1 and Snail2, and nuclear markers: β-catenin. In 2015, researchers from Shanghai Jiao Tong University have confirmed that N-cadherin is a novel prognostic biomarker to monitor the EMT progression of colorectal cancer (Yan et al., 2015). Similarly, in 2018, another group of researchers from Nanjing, China further validated that N-cadherin may also be a biomarker for EMT in laryngeal squamous cell carcinoma (Zhu et al., 2018). Therefore, reported in multiple cancer subtypes, N-cadherin is definitely an effective biomarker for EMT monitoring. As for *FSP1*, early in 1997, researchers confirmed the specific role of *FSP1* for triggering EMT at its early stage, implying *FSP1* as an effective biomarker for EMT (Okada et al., 1997). As for α-SMA, in 2017, a systematic summary of EMT during pancreatic cancer tumorigenesis revealed the specific role of α-SMA for epithelial to mesenchymal transformations (Aiello et al., 2017). For the extracellular matrix proteins, collagens like α1(I,III) collagens have been reported to participate in the epithelial to mesenchymal transformations during renal fibrosis (Zeisberg et al., 2001) and squamous cell carcinoma (Scanlon et al., 2013). Transcription factors: Snail1 and Snail2 have also been reported to directly regulate E-cadherin related pathways, further participating in epithelial to mesenchymal transformation. Apart from that, nuclear markers, such as β-catenin (Gu et al., 2016), have also been systematically identified to be associated with EMT. Conventional studies focused on tumor EMT progression on the whole tissue level. However, tissues comprise various cell types, including epithelial and mesenchymal cells. Therefore, such biomarkers can only define and recognize large tissues with obvious EMT markers but cannot accurately monitor the detailed EMT processes. With the development of single-cell sequencing techniques, gene expression profiling at single-cell resolution can be easily obtained. A previous study (Kiemer et al., 2001) used a spontaneous cancer model to monitor EMT progression. YFP + Epcam+ TCs are defined as epithelial-like cells, and YFP+ Epcam- TCs are defined as mesenchymal-like cells. In this study, we performed single-cell RNA sequencing on these single cells and revealed their detailed expression profiling to construct an expression profiling atlas for EMT progression at the single-cell level.

In this study, using such public expression profiling data and our newly constructed computational methods, we firstly detected the typical expression patterns of epithelial and mesenchymal TCs. We then identified signatures that can distinguish two cell groups at the single-cell transcriptomic level. In contrast to previous studies focusing on tissue characteristics, we precisely identified new signatures for distinguishing single-cell isolated TCs with unique epithelial or mesenchymal markers. Therefore, our discovered gene signatures may provide an effective and precise transcriptomic reference to monitor EMT progression at the single-cell level. Results contribute to the exploration of detailed tumorigenesis mechanisms during EMT.

## Materials and Methods

### Dataset

We obtained the mouse single-cell gene expression profiles of 71 epithelial YFP + Epcam + skin squamous cell carcinoma TCs and 312 mesenchymal-like YFP + Epcam − skin squamous cell carcinoma TCs from the study of Pastushenko et al. (2018) at https://www.ncbi.nlm.nih.gov/geo/query/acc.cgi?acc=GSE110357. Epithelial YFP + Epcam + TCs and mesenchymal-like YFP + Epcam − TCs represent different EMT states. Each TC was encoded with the expression levels of 49,585 genes. Expression differences may reveal the cascade mechanisms of tumor migration and invasion.

### Feature Selection

Feature selection aims to obtain specific features (i.e., gene signatures) for distinguishing epithelial TCs from mesenchymal TCs by using single-cell data. In this study, we used Boruta feature selection and minimum redundancy maximum relevance (mRMR) method (Peng et al., 2005) to evaluate the importance of each feature. We then selected key features, which were fed into the incremental feature selection (IFS) (Zhang et al., 2016; Lei et al., 2018; Li and Huang, 2018; Zhang et al., 2018; Chen et al., 2019; Li et al., 2019; Pan et al., 2019) with supervised classifiers to identify the optimal gene signatures for screening different TCs.

#### Boruta Feature Selection

Boruta feature selection (Kursa and Rudnicki, 2010) is a wrapper method based on random forest (RF) for detection of all relevant features associated with target outputs. This method iteratively identifies relevant features by comparing the importance scores of real and shuffled features. Boruta first copies the training dataset and shuffles the values of individual features, in which the new dataset is called shuffled dataset. A RF classifier is trained on this shuffled dataset, and the importance score for each feature is calculated. Boruta evaluates the importance score of individual features in the original training dataset and keeps the real features with remarkably higher importance scores than shuffled features. After multiple iterations, Boruta finally selects all the relevant features, and these features are further analyzed by the mRMR method.

#### Minimum Redundancy and Maximum Relevance

To select a refined feature set with good classification effects, mRMR (Peng et al., 2005) tries to balance the relevance between feature and target and the redundancy between features.

Considering that the features can be highly correlated, the combination of individual good features does not increase the classifier performance, leading to the redundancy of features. The solution proposed by mRMR involves measurement of feature correlation and resolve the redundancy between features. mRMR can maximize the correlation between features and target variable (maximum relevance), while minimizing the correlation between features (minimum redundancy). Mutual information (MI) is utilized to evaluate the correlation between features or target variable, and it is defined as follows:

(1) $$I\,\left(x,y\right)=\iint p\,\left(x,y\right)\,\log\frac{p\,\left(x,y\right)}{p\,\left(x\right)\,p\,\left(y\right)}\,d\,x\,d\,y$$

where the marginal probabilistic density of *x* and *y* is defined as *p*(*x*) and *p*(*y*), and the joint probabilistic density of *x* and *y* is represented by *p*(*x*, *y*). Accordingly, a ranked feature list obeying the criteria of mRMR can be constructed. In detail, several selection rounds are performed. Each round selects a feature with maximum correlation to target variable and minimum correlation to already-selected features. The ranked feature list sorts features according to their selection orders.

#### Incremental Feature Selection

Incremental feature selection is a feature selection method with a supervised classifier to detect optimal features, which are used to accurately distinguish the class labels corresponding to different samples (Cai et al., 2012; Zhang et al., 2015; Zhou et al., 2016; Chen et al., 2017a,b). To perform IFS in the above mRNR-ranked feature list, we first created a series of feature subsets by iteratively adding top-ranked features into the candidate feature subsets and then testing all feature subsets by building their classifiers. The subset of features with the optimal classification performance was finally obtained.

### Classification Learning

#### Support Vector Machine

Support vector machine (SVM) is a supervised learning algorithm based on statistical learning theory. It uses kernel techniques to map data from low-dimensional non-linear space to high-dimensional linear space, and then fits linear functions for new data in such high-dimensional space. The SVM infers the hyperplane with the largest margin between the two classes of samples. In this study, we used the sequence minimization optimization (SMO) algorithm to train the SVM, and the popular machine learning algorithm software, Weka, was employed for the classifier “SMO.”

#### Random Forest

Random forest is a supervised classifier constructed by a large number of decision trees, and it is mainly used to establish a classification prediction model and has been widely used in biological data (Pan et al., 2010). By summarizing votes from different decision trees, tree classifiers in the RF can determine their output categories, although each decision tree might have some differences from other decision trees. Considering the overfitting problem, the average prediction values of all decision trees are applied to avoid over-learning, which can reduce the prediction variance although it slightly increases the prediction bias.

#### Rule Learning

In this study, repeated incremental pruning to produce error reduction (RIPPER) was applied to produce classification rules for classifying samples from different TCs, where RIPPER can learn interpretable classification (e.g., IF-ELSE rules) for making prediction on new data.

### SMOTE

As mentioned in section “Dataset,” the abundance of mesenchymal TCs is much more than the epithelial TCs by approximately 4.4 times. Results indicated that the dataset consisting of these cells was imbalanced. When building the classifier, we used the synthetic minority over-sampling technique (SMOTE) to tackle this problem. This method generates new samples and pours them into the minor class. In detail, one sample, say *x*, is randomly selected from the minor class, its Euclid distance to other samples in this class is calculated, and the *k* nearest neighbors are selected. Then, one neighbor, say *y*, is randomly selected. A new sample is produced by the linear combination of *x* and *y*. After generating a predefined number of samples, the size of minor class can be equal to that of major class. In this study, we used the tool “SMOTE” in Weka to generate new samples and poured them into the class of epithelial TC. Finally, the numbers of epithelial tumor and mesenchymal TCs are equal.

### Performance Measurement

To estimate the performance measurement, we employed Matthew’s correlation coefficient (MCC), which is calculated by the discretization of binary variable. In the application of 10-fold cross-validation, the MCC formula used to evaluate the performance of the training model is as follows:

(2) $$M\,C\,C=\frac{T\,P\times T\,N-F\,P\times F\,N}{\sqrt{\left(T\,P+F\,P\right)\,\left(T\,P+F\,N\right)\,\left(T\,N+F\,P\right)\,\left(T\,N+F\,N\right)}}$$

where TP and FP represent the number of true-positive and false-positive predictions, respectively. TN represents the number of true-negative predictions, and FN represents the number of false-negative predictions.

### Functional Enrichment Analyses

For systematically investigate the set of genes that separate epithelial and mesenchymal cell status, we performed functional enrichment analyses (gene ontology enrichment analyses and KEGG pathway enrichment analyses) for the optimal predicted genes (237 genes obtained by optimal feature selection) using DAVID website^1^. The threshold for Benjamini–Hochberg adjusted *p*-value (FDR) was set at 0.05.

## Results

In this study, we first used Boruta to select relevant features, resulting in 237 features, which are listed in Supplementary File 1. The 237 remaining features were used as input into the mRMR method to generate a ranked feature list (Supplementary File 1).

**FIGURE 1:**
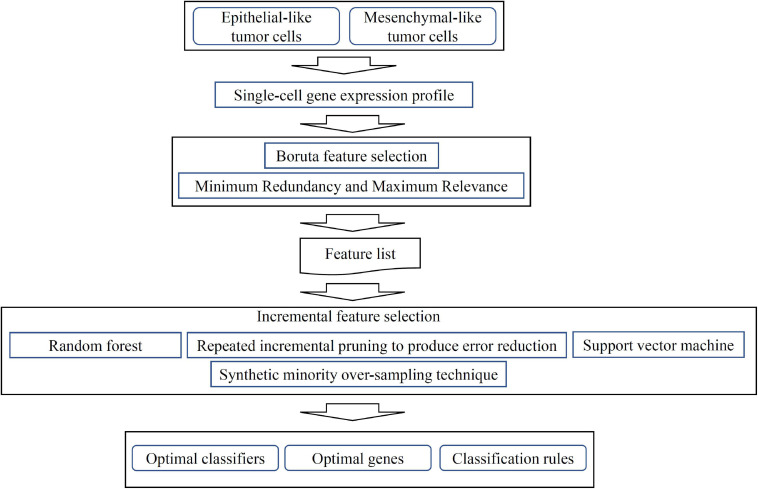
Flow chart of computational analysis. A systematic workflow integrating feature selection, machine learning models, and rule learning was applied to identify potential biomarkers for distinguishing single-cell isolated tumor cells (TCs). Optimal classifiers, genes and rules were identified based on the performance of different machine learning, rule learning models and the importance of features in each model.

**FIGURE 2:**
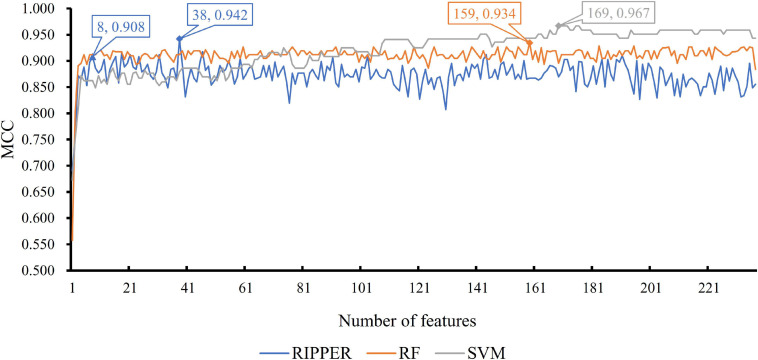
Incremental feature selection (IFS) curve of different classifiers. For each curve corresponding to one method, two performance peaks are annotated to indicate the number of optimal features selected and the accuracy of their classification model.

We then ran the IFS with SVM, RIPPER, and RF, respectively, on the generated candidate subset of features in terms of the feature list to determine the optimal features for classifying different TCs (Figure 1). As shown in Table 1 and Figure 2, SVM yielded the largest MCC value of 0.967. For RF, the largest MCC value obtained was 0.934. If we used IFS with RIPPER, we could obtain the largest MCC value of 0.942. Hence, SVM exhibits the best performance. However, SVM and RF are “black-box” methods. Although RIPPERT yields a slightly lower MCC than SVM, it can generate the interpretable classification rules. The performance corresponding to individual feature subsets by different classifiers is provided in Supplementary File 2.

**TABLE 1:** Performance of incremental feature selection (IFS) with support vector machine (SVM), random forest (RF), and repeated incremental pruning to produce error reduction (RIPPER) for classifying different tumor cells.

**Classifier**	**Number of features**	**SN**	**SP**	**ACC**	**MCC**
SVM	169	1.000	0.987	0.990	0.967
RF	159	0.986	0.978	0.979	0.934
RIPPER	38	0.986	0.981	0.982	0.942

As previously mentioned, although the classification rules generated by RIPPER provide lower performance than the two other classifiers, these rules can provide more interpretable information. By checking the trend of MCC yielded by the IFS with RIPPER, when the top eight features are used, the rules yielded by RIPPER can result in a satisfactory MCC of 0.908, which is only 3.4% lower than the best MCC of RIPPER. Thus, we used these eight features to generate rules by using RIPPER, thus obtaining three classification rules, which are listed in Table 2.

**TABLE 2:** Three classification rules learned by repeated incremental pruning to produce error reduction (RIPPER).

**Index**	**Rule**	**Tumor cell type**
Rule 1	ENSMUSG00000045394 (*Epcam*) < = 47.481	Mesenchymal tumor cell
Rule 2	ENSMUSG00000031565 (*Fgfr1*) > = 130.294 and ENSMUSG00000051397 (*Tacstd2*) < = 14.977	Mesenchymal tumor cell
Rule 3	Others	Epithelial tumor cell

For functional enrichment analyses, we found multiple GO terms and KEGG pathways like GO:0007155 (cell adhesion), GO:0005576 (extracellular region), and ECM-receptor interaction (KEGG pathways). Complete results for functional enrichment analyses were shown in Tables 3–6.

**TABLE 3:** Gene ontology enrichment results, biological processes (BP).

**GO term**	**Description**	***P*-value**	**FDR**	**GO cluster**
GO:0007155	Cell adhesion	1.1E-15	1.5E-12	BP
GO:0042060	Wound healing	5.6E-11	0.000000039	BP
GO:0043588	Skin development	1.1E-09	0.00000049	BP
GO:0043616	Keratinocyte proliferation	2.3E-07	0.000075	BP
GO:0030199	Collagen fibril organization	2.7E-07	0.000075	BP
GO:0030198	Extracellular matrix organization	7.9E-07	0.00018	BP
GO:0061436	Establishment of skin barrier	0.000002	0.00039	BP
GO:0001501	Skeletal system development	0.000004	0.0007	BP
GO:0002009	Morphogenesis of an epithelium	0.0000085	0.0013	BP
GO:0035987	Endodermal cell differentiation	0.000013	0.0018	BP
GO:0007156	Homophilic cell adhesion via plasma membrane adhesion molecules	0.00012	0.015	BP
GO:0001568	Blood vessel development	0.00017	0.019	BP
GO:0010482	Regulation of epidermal cell division	0.00039	0.039	BP
GO:0060672	Epithelial cell morphogenesis involved in placental branching	0.00039	0.039	BP
GO:0001775	Cell activation	0.0005	0.046	BP

**TABLE 4:** Gene ontology enrichment results, cellular components (CC).

**GO term**	**Description**	***P*-value**	**FDR**	**GO cluster**
GO:0005576	Extracellular region	6.3E-20	8E-18	CC
GO:0031012	Extracellular matrix	6.4E-20	8E-18	CC
GO:0070062	Extracellular exosome	9.8E-20	8.2E-18	CC
GO:0031012	Proteinaceous extracellular matrix	4.5E-16	2.8E-14	CC
GO:0005615	Extracellular space	8E-11	3.8E-09	CC
GO:0005604	Basement membrane	9.1E-11	3.8E-09	CC
GO:0005581	Collagen trimer	3E-09	0.00000011	CC
GO:0030057	Desmosome	0.000011	0.00033	CC
GO:0009986	Cell surface	0.000014	0.00039	CC
GO:0005912	Cell–cell adherens junction	0.00002	0.00049	CC
GO:0030054	Cell junction	0.000032	0.00073	CC
GO:0005925	Focal adhesion	0.000053	0.0011	CC
GO:0005882	Intermediate filament	0.00042	0.0081	CC
GO:0005610	Laminin-5 complex	0.00078	0.014	CC
GO:0005887	Integral component of plasma membrane	0.0013	0.022	CC
GO:0016020	Membrane	0.0014	0.022	CC
GO:0016323	Basolateral plasma membrane	0.0025	0.037	CC

**TABLE 5:** Gene ontology enrichment results, molecular functions (MF).

**GO term**	**Description**	***P*-value**	**FDR**	**GO cluster**
GO:0050840	Extracellular matrix binding	2.5E-08	0.0000083	MF
GO:0048407	Platelet-derived growth factor binding	1.5E-07	0.000022	MF
GO:0008201	Heparin binding	0.0000002	0.000022	MF
GO:0005201	Extracellular matrix structural constituent	0.0000074	0.00061	MF
GO:0005509	Calcium ion binding	0.000023	0.0014	MF
GO:0098641	Cadherin binding involved in cell–cell adhesion	0.000024	0.0014	MF
GO:0005198	Structural molecule activity	0.0001	0.0049	MF
GO:0050839	Cell adhesion molecule binding	0.00018	0.0073	MF
GO:0005515	Protein binding	0.00023	0.0077	MF
GO:0005044	Scavenger receptor activity	0.00023	0.0077	MF
GO:0005518	Collagen binding	0.00061	0.018	MF
GO:0005507	Copper ion binding	0.00071	0.02	MF
GO:0030169	Low-density lipoprotein particle binding	0.00078	0.02	MF
GO:0016641	Oxidoreductase activity, acting on the CH-NH2 group of donors, oxygen as acceptor	0.0013	0.031	MF

**TABLE 6:** KEGG pathway enrichment results.

**Description**	***P*-value**	**Benjamini FDR**
ECM-receptor interaction	5.9E-10	8.4E-08
Protein digestion and absorption	0.0000012	0.000083
Focal adhesion	0.0000081	0.00038
Amoebiasis	0.000013	0.00045
PI3K-Akt signaling pathway	0.000093	0.0026
Ribosome	0.0004	0.0095
Proteoglycans in cancer	0.00086	0.017

## Discussion

As previously mentioned, by using our newly presented computational approaches, we screened out a group of effective genes and their expression rules that can distinguish epithelial and mesenchymal TCs at the single-cell transcriptomic level. Such optimal genes and rules have already been validated by recent publications, and their detailed analyses are summarized below.

### Optimal Genes for EMT Cell Clustering

The first identified gene in our prediction list was *Vim* (ENSMUSG00000026728). It is a protein-coding gene that participates in cellular signaling transduction and cell proliferation (Pekny et al., 1999; Malanchi et al., 2011). For its specific role in distinguishing epithelial and mesenchymal cells, a specific study published in 2015 reported that the expression level of our predicted gene, *Vim*, is significantly upregulated after EMT processes (Fischer et al., 2015). Therefore, epithelial and mesenchymal TCs may have quite different expression levels of such gene, corresponding with our prediction. Moreover, this gene is a biomarker of EMT (Seton-Rogers, 2016; Wang et al., 2016), thereby validating its specific expression pattern in epithelial and mesenchymal cells.

Another predicted gene, namely, *Bgn* (ENSMUSG00000031375), is differentially expressed before and after EMT. This gene mainly participates in carbohydrate derivative binding and metabolisms (Bartlett and Park, 2010). During tumorigenesis, this gene participates in EMT both in human beings and mouse models (Summers et al., 2010; Anastassiou et al., 2011). In mouse models, during EMT, *Bgn* stimulates the synthesis of fibrillin-1 and participates in connective tissue regulation (Schaefer et al., 2004), thus remodeling the related microenvironment. Although no direct reports have confirmed that *Bgn* contribute to the remodeling of the tumor microenvironment and participate in EMT for tumorigenesis, various evidence of Bgn in multiple mouse disease models (Chen et al., 2000; Schaefer et al., 2004; Cheng et al., 2012; Sugg et al., 2014) have confirmed its different pathological significance. This finding indicates that *Bgn* has a distinctive expression pattern before and after EMT, thereby validating our prediction. As for the specific contribution of such gene at single cell level, according to a recent publication on *Cancer Cell* (Zhou et al., 2020), such gene has also been shown to participate in EMT during tumorigenesis of colorectal cancer at a single cell level.

*Epcam* (ENSMUSG00000045394) is the major distinctive marker for initial cell sorting. The identification of such gene in our optimal prediction list not only validated the efficacy of the original cell sorting processes but also confirmed the accuracy of our prediction. As a famous cell surface marker, *Epcam* participates in the regulation of cell differentiation, proliferation, and death (Munz et al., 2004, 2009). For the distinctive expression pattern of *Epcam* in epithelial and mesenchymal TCs, its protein product is the EPCAM cellular surface protein, which is one of the most classical molecular biomarkers for distinguishing epithelial and mesenchymal cells. Therefore, at the transcriptome level, such a gene definitely has different expression levels in epithelial and mesenchymal TCs (Thiery and Lim, 2013; Pastushenko et al., 2018; Pastushenko and Blanpain, 2019).

We also identified *Serinc2* (ENSMUSG00000023232) as distinctive marker, which participates in lipid metabolism (Lee et al., 2008; Sanderson et al., 2010). This gene has alternative expression patterns during tumorigenic degeneration and EMT (Alibardi, 2019). This gene is also functionally connected to a famous regulator for EMT and TGF-beta (Kasai et al., 2005; Shen et al., 2015). Therefore, considering the specific role of TGF-beta during EMT (Kasai et al., 2005), the expression level of Serinc2 as the downstream of TGF-beta (Kasai et al., 2005) may be altered during EMT progression, validating the efficacy and accuracy of our prediction.

We also identified the *Fgfr1* gene (ENSMUSG00000031565), which has different expression patterns in epithelial TCs compared with mesenchymal TCs. As a regulator for cell differentiation, proliferation, and adhesion, the *Fgfr1* gene is one of the major regulatory genes for EMT in multiple cancer subtypes (Nguyen et al., 2013; Ware et al., 2013; Jiao et al., 2015). For its detailed expression alteration during EMT, regulated by microRNA-198, the upregulation of *Fgfr1* and its ligand *FgF1* may promote the EMT processes (Mori et al., 2015). Therefore, *Fgfr1* may have quite different expression levels before and after EMT (Mori et al., 2015).

We also identified the *Fxyd3* gene (ENSMUSG00000057092), which is functionally related to cell adhesion as a part of TGF-beta signaling pathway (Okudela et al., 2009; Widegren et al., 2009). This gene has different expression levels in breast cancer cells with different proliferative and differential potentials, validating the distinctive expression levels of Fxyd3 in different cancer cell subtypes (Xue et al., 2019). Moreover, some studies on TGF-beta signaling pathway confirmed that *Fxyd3*, as a negative TGF-beta signaling regulator, induces EMT (Yamamoto et al., 2011), indicating its potential distinctive expression levels before and after EMT.

We also identified the *Fstl1* gene (ENSMUSG00000022816), which is a regulator of embryo development and cell differentiation (Geng et al., 2011; Xu et al., 2012). During the transformation of TCs from epithelial status to mesenchymal status, Fstl1 regulates the complexity of cellular junctions, thereby promoting EMT (Zuo et al., 2011). For the differential expression pattern of *Fstl1* in epithelial and mesenchymal TCs, the expression level of this gene is positively related to the pathogenic results of EMT (Gu et al., 2018). Hence, *Fstl1* may be upregulated in mesenchymal TCs. Apart from that, such gene has also been shown to be a single-cell level EMT biomarker. Although no other single cell level studies identified such gene as EMT biomarkers, such gene has been detected to be associated with EMT transformation based on breast cancer cells in vitro cultured from single-cell suspension, indicating that the EMT of some cells in breast cancer may be associated with this gene.

The last predicted gene in our optimal gene list is *Tacstd2* (ENSMUSG00000051397). As a specific calcium signal transducer, this gene participates in cell differentiation (Eisenwort et al., 2011). Similar to Epcams, *Tacstd2* is a typical biomarker for cells with epithelial phenotypes (Eisenwort et al., 2011). In addition, *Tacstd2* is a typical marker for TCs with epithelial characteristics different from EMT-transformed mesenchymal TCs (Chen et al., 2013). Therefore, *Tacstd2* can be screened as a specific signature to distinguish epithelial and mesenchymal cells, validating the efficacy and accuracy of our prediction. Specifically, such gene has been identified as a single cell level EMT biomarker in pancreatic cancer stem-like cells, which cannot be directly identified using bulk sequencing (Bao et al., 2014).

### Optimal Rules for EMT Cell Clustering

Apart from the above qualitative analyses on optimal genes that may distinguish epithelial and mesenchymal TCs at the single-cell transcriptome level, we also identified a group of quantitative rules that may further accurately distinguish and interpret such two cell groups on the basis of the detailed cell clustering and establishment of related measurement standards. According to our prediction results, the top three rules can distinguish two groups of TCs with the best performance. Two rules can distinguish two clusters of mesenchymal TCs from epithelial TCs. The first rule we screened only involved *Epcam* (ENSMUSG00000045394). According to the rule, cells with *Epcam* expression level lower than 47.481126 FPKM are mesenchymal TCs. This finding is reasonable because Epcam is the golden standard for identification of epithelial and mesenchymal cells. As for the threshold, according to the mouse genome database (Bult et al., 2019), in most epithelial tissues, the expression level of such gene is higher than 50 FPKM. Hence, we can easily distinguish epithelial and mesenchymal cells with such threshold, validating the efficacy and accuracy of our prediction.

For the second rule, cells with high level of *Fgfr1* (ENSMUSG00000031565) and low level of *Tacstd2* (ENSMUSG00000051397) are mesenchymal TCs. The high level of *Fgfr1* promotes the EMT and remains in the mesenchymal status (Thiery and Lim, 2013; Pastushenko et al., 2018; Pastushenko and Blanpain, 2019), while *Tacstad2* is downregulated similar to *Epcam*, indicating that such cells may be mesenchymal cells (Chen et al., 2013), validating the accuracy of our prediction. Therefore, the combination of the two supporting parameters can accurately identify mesenchymal cells, validating the accuracy of our prediction.

### GO Enrichment and KEGG Pathway Analyses on Optimal Genes for EMT Cell Clustering

For systematically investigation on the functional distribution of optimal genes, we performed GO enrichment and KEGG pathway enrichment analyses on such optimal genes. As we have described in Tables 3–6, with the FDR threshold as 0.05, we identified 15 biological processes, 17 cellular components, 14 molecular functions, and seven KEGG pathways with our optimal genes enriched. Here, we chose the top enriched GO terms and KEGG pathways in each cluster for detailed analyses.

For GO enrichment results of biological process levels, the first identified GO term, GO:0007155 (cell adhesion) has been shown to enrich optimal genes. According to recent publications, cell–cell adhesion has been shown to be linking Wnt/β-catenin signaling pathway with EMT (Basu et al., 2018) and interacting with extracellular matrix, promoting EMT (Kumar et al., 2014). Therefore, it is reasonable for our optimal genes to enrich in such GO term. Apart from that, GO:0042060 (wound healing) and GO:0043588 (skin development) have also been shown to enrich optimal genes associated with EMT. According to recent publications, both wound healing which involves cell–cell and cell-extracellular matrix interactions (Kalluri, 2009; Yin et al., 2013) and skin development which associates with TGF-β signaling pathways (Liarte et al., 2020) have been reported to be functionally related to the transformation between epithelial and mesenchymal cells (Kalluri, 2009; Yin et al., 2013; Liarte et al., 2020), validating our prediction.

As for GO enrichment results of cellular components level, apart from general terms like GO:0016020 (membrane) and GO:0009986 (cell surface), we also identified some specific terms that may reflect the functional enrichment pattern of the optimal genes. GO:0005912 (cell–cell adherens junction) and GO:0005925 (focal adhesion) are two enriched GO terms in this cluster, both of which are directly related with cell adhesion that we have discussed above to be associated with EMT. The top five GO terms GO:0005576 (extracellular region), GO:0031012 (extracellular matrix), GO:0070062 (extracellular exosome), GO:0031012 (proteinaceous extracellular matrix), and GO:0005615 (extracellular space) are all associated with extracellular components and their interactions with cells. As we have discussed above, cell-extracellular interactions are quite fundamental for EMT (Sangaletti et al., 2008; Gibbons et al., 2009). Therefore, the enrichment of our optimal genes in such cluster may further validate the efficacy and accuracy of our prediction.

Multiple molecular function GO terms have also enrich our optimal genes. Apart from cell adhesion and extracellular matrix associated GO terms like GO:0050840 (extracellular matrix binding), GO:0050839 (cell adhesion molecule binding), and GO:0098641 (cadherin binding involved in cell–cell adhesion), we also identified functional GO terms GO:0008201 (heparin binding) and GO:0005507 (copper ion binding). According to recent publications, heparin binding has been shown to EMT by linking the TGF-β signaling pathways (Kantola et al., 2008). As for copper ion binding, as reported in 2012, researchers have confirmed that iron chelators have regulated the TGF-β signaling pathways via copper binding associated biological processes (Chen et al., 2012), validating the enrichment results.

As for KEGG pathways, ECM-receptor interactions and focal adhesions have been enriched. As we have described above, focal adhesion and extracellular matrix has been validated to be associated with EMT, further validating our results. Apart from that, PI3K-Akt signaling pathway has also been confirmed to enrich EMT associated genes. According to recent publications, such results have also been validated (Xu et al., 2015; Wei et al., 2016).

## Conclusion

All our identified key features (genes and rules) were validated to participate in the qualitative and quantitative clustering of different TCs, reflecting different stages of EMT progression. Therefore, our computational approach may be an effective method to identify specific gene signatures for clustering different TC subgroups at the single-cell level. This work provides a new tool for elucidating the detailed regulatory mechanisms of tumor EMT progression.

## Data Availability Statement

Publicly available datasets were analyzed in this study. This data can be found here: https://www.ncbi.nlm.nih.gov/geo/query/acc.cgi?acc=GSE110357.

## Author Contributions

XY, TH, and Y-DC conceived the concept of the work. XP and LC performed the experiments. XP, XY, SZ, Y-HZ, and SW made analysis. XY and XP prepared the draft. XY, XP, and TH revised the manuscript. All authors contributed to the article and approved the submitted version.

## Conflict of Interest

The authors declare that the research was conducted in the absence of any commercial or financial relationships that could be construed as a potential conflict of interest.

## Footnotes

1 https://david.ncifcrf.gov/

## Supplementary Material

Supplementary File 1 Relevant features yielded by Boruta feature selection method and their ranks obtained by the mRMR method.

Supplementary File 2 Performance of IFS corresponding to different number of features used.
